# Hashimoto’s thyroiditis reduces central lymph node metastasis risk in papillary thyroid microcarcinoma: an integrated meta-analysis

**DOI:** 10.3389/fendo.2025.1695508

**Published:** 2025-11-24

**Authors:** Linkun Zhong, Changlian Xie, Xiaoxiong Gan, Jianhang Miao, Yaming Wu, Yunyun Yang, Chizhuai Liu, Yutong Li

**Affiliations:** 1 The Department of General Surgery, Zhongshan City People’s Hospital, Zhongshan, Guangdong, China; 2 Medical School, Shenzhen University, Shenzhen, Guangdong, China; 3 The Intensive Care Unit, Zhongshan Hospital of Traditional Chinese Medicine Affiliated to Guangzhou University of Chinese Medicine, Zhongshan, Guangdong, China; 4 Department of Thyroid Surgery, Guangzhou First People’s Hospital, School of Medicine, South China University of Technology, Guangzhou, Guangdong, China; 5 The Department of General Surgery, Xuwen County People’s Hospital, Zhanjiang, Guangdong, China; 6 The First School of Clinical Medicine, Guangdong Medical University, Zhanjiang, Guangdong, China

**Keywords:** lymphatic metastasis, papillary carcinoma of thyroid, Hashimoto disease, fine-needle aspiration biopsies, clinically node-negative (cN0)

## Abstract

**Background:**

Hashimoto’s thyroiditis (HT) is the most common comorbidity in patients with papillary thyroid microcarcinoma (PTMC). The necessity of prophylactic central lymph node dissection (CLND) in clinically node-negative (cN0) PTMC cases remains a topic of debate. This study evaluates the risk of cervical lymph node metastasis (LNM) in PTMC patients with concurrent HT.

**Objectives:**

This study aims to evaluate the risk of central lymph node metastasis (CLNM) in patients with PTMC concurrent with HT. By synthesizing existing literature and conducting a case–control analysis, we seek to enhance individualized risk assessment and inform surgical decision-making for PTMC patients.

**Methods:**

We conducted a search for studies published before 1 June 2025 that assessed the risk of CLNM in PTMC concurrent with HT on PubMed, Embase, and Web of Science. A total of 17 studies involving 11,873 cases were included in this meta-analysis. Additionally, we performed a case–control study through a retrospective analysis of 303 consecutive PTMC patients who underwent surgery between 2017 and 2024.

**Results:**

The meta-analysis indicated that HT was present in 3,175 of the 11,873 PTMC cases (26.7%). The rate of positive CLNM was significantly lower in the HT group (32.6%) compared to the non-HT group (38.4%), with an odds ratio of 0.75. The false-negative rate was as low as 27.5% when combining ultrasonography (US) and fine-needle aspiration biopsy (FNAB) to evaluate CLN status. Funnel plots showed no significant publication bias. In the retrospective analysis, the CLN examination rate in the HT group was significantly higher than in the non-HT group, yet the incidence of CLNM was lower in the HT group. ROC curve analysis indicated that the TPOAb cutoff point for CLNM was 17.9, with sensitivity and specificity values of 53% and 68%, respectively.

**Conclusion:**

HT may reduce the risk of CLNM in patients with PTMC, suggesting a protective role. Predictive, preventive, and reliable preoperative evaluations using ultrasound and FNAB enhance the assessment of lymph node status, with TPOAb serving as an important marker. These insights support the development of personalized strategies for early intervention and improved patient management in PTMC.

**Systematic Review Registration:**

https://www.crd.york.ac.uk/PROSPERO/, identifier CRD420251174681.

## 1 Introduction

Papillary thyroid cancer (PTC) accounts for 85% to 90% of all thyroid carcinomas and has seen a rising incidence in recent years, particularly in the case of papillary thyroid microcarcinoma (PTMC). This increase may be attributed to recent advancements in diagnostic imaging techniques, such as high-resolution ultrasonography (HR-US) (1), computed tomography (CT), and magnetic resonance imaging (MRI), which have enhanced the detection rates of early-stage PTC (2). PTC with a diameter less than or equal to 1 cm is defined as PTMC (3). Although the overall prognosis of PTMC patients is very excellent, lymph node metastases (LNM), especially central LNM (CLNM), are common, occurring in 12%–64% of patients (4). There is more controversy about whether prophylactic central lymph node dissection (PCND) should be performed in PTMC patients with clinical lymph node-negative (cN0). However, few studies suggest that PCND can reduce the recurrence rate and improve the survival rate of PMTC patients (5, 6). The most important morbidities associated with CLND consist of recurrent laryngeal nerve damage and hypocalcemia related to parathyroid hypofunction or accidental parathyroidectomy (7).

Hashimoto’s thyroiditis (HT) is a common autoimmune disease in which the immune system attacks the thyroid gland (8, 9). Dailey et al. first proposed the association between HT and PTC in 1955 (10). Previous studies have reported that the incidence rate of PTC concurrent with HT ranges from 0.3% to 38% (11, 12). Simultaneously, some studies have reported that a high proportion (40%–80%) of cN0 PTMC patients were changed to clinically positive after undergoing prophylactic CLND (13). However, another study proposed that PTC concurrent with HT has been less aggressive with earlier presentation and fewer chances of extrathyroidal extension and LNM (14). According to the current ATA guidelines, prophylactic CLND is not recommended for PTMC patients with cN0 status. However, accurately assessing CLN status can be challenging, especially in patients with concurrent HT. Furthermore, existing studies on PTMC patients with HT are often limited to single institutions and involve relatively modest sample sizes. Current American Thyroid Association (ATA) guidelines do not support prophylactic CLND for cN0 PTMC patients.

We hypothesize that the identification of TPOAb as a biomarker for CLNM will facilitate risk stratification and tailored interventions. Recent studies have shown that TPOAb levels correlate with LNM in PTMC, highlighting their potential as predictive markers (15–17). Furthermore, the integration of advanced imaging techniques and molecular profiling could improve the accuracy of preoperative assessments (18, 19). This study integrated meta-analysis and retrospective analysis to investigate the risk of cervical LNM in patients with PTMC concurrent with HT, as well as the diagnostic efficacy of TPOAb for identifying CLNM. Our meta-analysis synthesizes data from multiple studies, enhancing predictive capabilities regarding lymph node involvement, while the retrospective analysis provides insights into TPOAb as a potential biomarker.

## 2 Methods

### 2.1 Data collection and eligibility criteria

**Figure 1:**
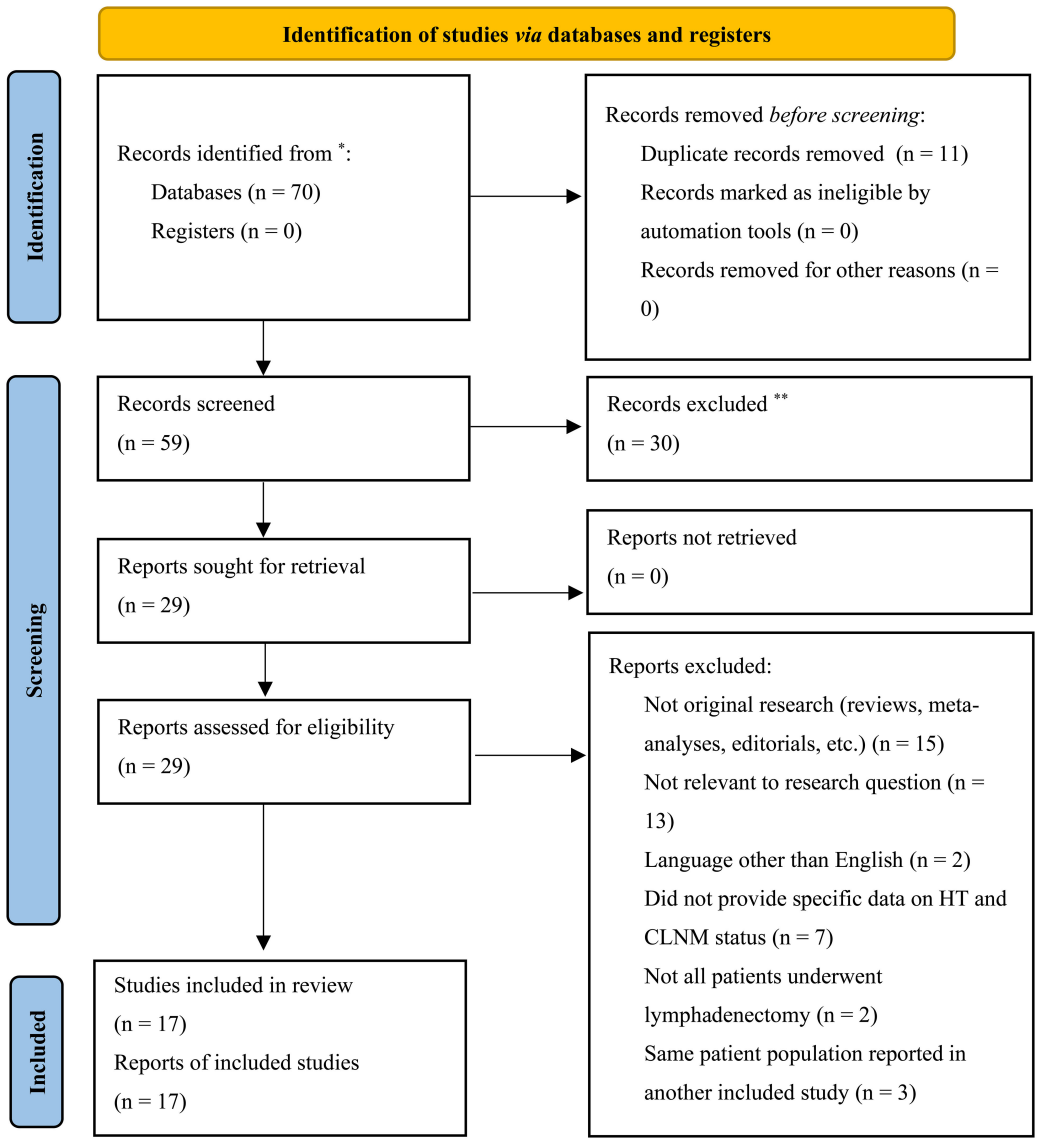
Search flow. ^*^Consider, if feasible to do so, reporting the number of records identified from each database or register searched (rather than the total number across all databases/registers). ^**^If automation tools were used, indicate how many records were excluded by a human and how many were excluded by automation tools.

We customized the individual search criteria. We searched PubMed, Embase, and Web of Science for relevant articles published before 1 June 2025. The following keywords were used: “Hashimoto disease OR HT OR Hashimoto’s syndrome OR Hashimoto’s struma OR chronic lymphocytic thyroiditis” and “thyroid cancer, papillary OR PTC.” The “related articles” function has expanded the scope of the search. If the same population was used in multiple published studies, we only extracted the most complete and recent one. The adopted literature need to meet the following criteria: 1) original articles demonstrated that the association between PTC and classical HT was assessed only in thyroid specimens by histopathologic examination; 2) all patients received thyroidectomy and lymphadenectomy; 3) articles published before 1 June 2025; 4) consisted of data on HT patients and the status of CLNM; and 5) HT needs to be confirmed by histopathological examination or the presence of thyroid antibodies, and LN pathologic status needs to been evaluated. Also, our studies excluded reviews, meta-analyses, editorials, case reports, meeting abstracts, and letters. Studies published in languages other than English were excluded. After the initial screening, we searched for the full text of the article (**Figure 1**).

### 2.2 Data extraction

Three investigators (ZLK, GXX, and YTL) independently extracted and summarized the data. Disagreements were resolved by consensus or by a third party. The extracted data included the first author, publication year, type of study design, country, number of patients, surgical strategy, clinical LN status, and methods used to evaluate LN.

### 2.3 Study quality assessment

The Newcastle-Ottawa Scale (NOS) was used to assess the quality of observational studies, including patient selection, study group comparability, and outcome assessment. Two investigators independently assessed each study using a rating scale of 0−9. Studies scoring six or more are considered high quality.

### 2.4 Statistical analysis

The chi-square test was used to evaluate statistical heterogeneity among the studies. An *I*² value of 50% or higher was considered indicative of high heterogeneity. In cases of high heterogeneity, a random-effects model was applied; otherwise, a fixed-effects model was utilized. Meta-analysis was performed using Review Manager 5.3 (Cochrane Collaboration, Oxford, U.K.). Dichotomous variables were analyzed using odds ratios (OR), and 95% confidence intervals (CIs) were calculated to estimate all the results. Publication bias was assessed qualitatively through visual inspection of the funnel plot.

We then compared the risk of CLNM between the cN0 group and the uncertain cN0 group in a subgroup analysis. For the purpose of this subgroup analysis, studies were categorized as follows: The cN0 group included studies where all patients were explicitly stated to be cN0 preoperatively. The uncertain cN0 group included studies that did not explicitly specify the preoperative clinical nodal status of their patient cohort, or studies that included a mix of cN0 and cN1 patients without providing separate data for the cN0 subgroup.

Additionally, subgroup analysis was performed between the US group, US+FNAB group, and US+CT+FNAB group to evaluate whether the risk of CLN metastasis was statistically different using different methods of evaluating LN status. Our study design follows the guidelines of the Preferred Reporting Items for Systematic Reviews and Meta-Analyses (PRISMA) and Assessing the Methodological Quality of Systematic Reviews (AMSTAR).

### 2.5 Data source

This retrospective study was approved by the Research Ethics Committee of Guangzhou First People’s Hospital. Written informed consent was obtained from all the participants. From January 2017 to December 2024, 303 consecutive patients with PTMC who underwent surgery at the Department of Thyroid Surgery, Guangzhou First People’s Hospital, and the Department of Thyroid Surgery, Zhongshan City People’s Hospital were enrolled in this study.

### 2.6 General information

A total of 76 men and 227 women aged between 15 and 79 years were included in the study. During the surgical procedure, non-PTMC was excised and subsequently confirmed histologically. Among the patients, 41 were diagnosed with HT, while 262 did not have this condition. The participants were categorized into two groups based on their HT status. Epidemiological data for these two groups are presented in **Table 1**. Out of the 133 patients who experienced LNM, 101 cases were classified as CLNM only, while 32 cases involved LLNM.

**Table 1:** Characteristics of the studies included.

First author	Year	Country	Study type	Surgery	cN0	Evaluated methods	Total cases	HT group	Non-HT group	Quality assessment
Yi-Li Zhou (21)	2012	China	CC	TT and P-CLND	Yes	US and FNAB	122	43	79	7
Bo-Yeon Kim (20)	2012	South Korea	CC	TT and P-CLND (bi)	Yes	US and FNAB	160	66	94	5
Yin-Long Yang (23)	2014	China	CC	TT and P-CLND (bi)	Yes	US, CT, and FNAB	291	92	199	6
H.Y. Bircan (22)	2014	Turkey	RE	Uncertain	Uncertain	FNAB	172	67	105	5
Li-Yang Zhang (34)	2015	China	C	TT and P-CLND (bi)	Yes	US and FNAB	178	42	136	5
Ying-Ying Xiang (26)	2015	China	CC	TT and P-CLND	Yes	US	392	85	307	7
Ning Qu (29)	2015	China	CC	TT and CLND	Uncertain	US	1,250	364	886	8
Ya-Wen Guo (25)	2015	China	CC	TT and P-CLND	Uncertain	NA	329	98	231	6
Husniye Baser (24)	2015	Turkey	RE	Uncertain	Uncertain	US and FNAB	491	162	329	6
L. Zhang (30)	2016	China	CC	TT and CLND	No	NA	1,226	293	933	6
Ki-Kim Seo (35)	2016	South Korea	C	TT and CLND and/or LLND	No	NA	5,137	1,488	3,649	8
Hua Liu (28)	2016	China	RE	TT and CLND	Uncertain	NA	168	49	119	6
Xing-Jian Lai (27)	2016	China	RE	Uncertain	Uncertain	US and FNAB	367	94	273	6
Xing-Jie Yin (32)	2017	China	CC	TT and CLND (bi)	Yes	US and FNAB	1,092	129	963	7
Yu-fei Wang (33)	2017	China	CC	TT and CLND and LLND	Uncertain	US	169	35	134	6
M. Li (36)	2017	China	C	TT and P-CLND (bi)	Yes	US	273	42	231	7
K. Osman (31)	2017	Turkey	CC	TT and P-CLND	Yes	US and FNAB	56	26	30	6

cN0, clinically node-negative; HT, Hashimoto’s thyroiditis; C, cohort; CC, case–control; RE, retrospective; TT, total thyroidectomy; CLND, central neck dissection; P-CLND, prophylactic central neck dissection; LLND, lateral neck dissection; cN0, clinically node-negative; Uncertain cN0, studies that do not mention evaluation of CLN status; US, ultrasonography; CT, neck computed tomography; FNAB, fine-needle aspiration biopsy.

### 2.7 Patient grouping and definition

The clinicopathological data between the two groups were compared, including age, gender, tumor foci, tumor size, extrathyroidal extension, lymphadenopathy, TNM stage, and 8th edition AJCC stage. Further analysis, including a cN0 subgroup analysis, was conducted to assess central/lateral neck lymph node status/metastasis and surgical indications in PTMC patients with and without HT.

### 2.8 Statistical analysis

Data were presented as mean ± standard deviation, and the receiver operating characteristic (ROC) curve and the area under the ROC curve were generated. All analyses were performed using GraphPad 8.3. Student’s *t*-test and the chi-square test were used to compare differences between the two groups. *P* < 0.05 was considered statistically significant.

## 3 Results

### 3.1 Characteristics of the studies

Seventy studies comprising 11,873 PTMC patients, including 3,175 with concomitant HT, met the inclusion criteria, including 10 case–control and three cohort studies (20–36) (**Table 2**). As a result, 32.6% (1,034/3,175) of patients with HT were diagnosed with CLNM. In contrast, 38.4% (3,340/8,698) of patients without HT developed CLNM. Seven of the 17 studies enrolled patients who underwent total thyroidectomy (TT) and CLND (21, 25, 26, 28–31). Five studies involved TT and bilateral CLND (20, 23, 32, 34, 36), while two studies involved lateral cervical lymph node dissection (LLND) (33, 35). Depending on the NOS, all studies met the inclusion criteria and were included in the meta-analyses: three studies were rated five stars, eight studies were rated six stars, four studies were rated seven stars, and two studies were rated eight stars.

**Table 2:** Univariate analyses of the association between HT and PTMC clinicopathological parameters in the total and cN0 groups.

Patients’ parameters	Total (303)	HT	Odds ratio	*P*-value	cN0 group (270)	HT	Odds ratio	*P*-value
	Non-HT				Non-HT			
Age	43.8 ± 12	41.5 ± 12	NA	0.248	44.1 ± 12	43.5 ± 13	NA	0.770
<55	209	33	1	0.553	183	27	1	0.536
≥55	53	8	0.965 (0.495–1.879)		52	8	1.033 (0.537–1.987)	
Sex								
Women	189	38	1	0.002*	169	32	1	0.008*
Men	73	3	0.263 (0.087–0.794)		66	3	0.305 (0.101–0.918)	
Tumor foci								
Unifocality	196	26	1	0.092	178	26	1	0.499
Multifocality	66	15	1.452 (0.923–2.286)		57	9	1.060 (0.578–1.945)	
ETE								
No	233	32	1	0.051	211	28	1	0.085
Yes	29	9	1.983 (1.013–3.881)		24	7	1.958 (0.913–4.202)	
Tumor size (mm)	6.7 ± 2.6	6.6 ± 2.3	NA	0.740	6.6 ± 2.6	6.5 ± 2.4	NA	0.791
T stage								
1–2	255	40	1	0.704	231	35	1	0.572
3–4	7	1	0.913 (0.115–7.229)		4	0	1.017 (1.000–1.035)	
Lymphadenopathy								
No	209	29	1	0.135	209	29	1	0.217
Yes	53	12	1.447 (0.849–2.466)		26	6	1.549 (0.687–3.495)	
N stage								
0	147	23	1	0.910	137	23	1	0.781
N1a	88	13	0.964 (0.606–1.535)		78	10	0.835 (0.483–1.443)	
N1b	27	5	1.151 (0.484–2.738)		20	2	0.628 (0.156–2.524)	
M stage								
M0	261	41	1	0.865	235	25	NA	NA
M1	1	0	1.004 (0.996–1.011)		0	0		
AJCC stage								
I–II	260	41	1	0.747	234	35	1	0.870
III–IV	2	0	1.008 (0.997–1.018)		1	0	1.004 (0.996–1.013)	

Mean ± standard deviation.

HT, Hashimoto’s thyroiditis; PTMC, papillary thyroid microcarcinoma; N0, clinically node-negative; ETE, extrathyroidal extension; T, tumor size; N, lymph node; M, metastasis; AJCC, 8th edition American Joint Committee on Cancer staging.

*P < 0.05.

### 3.2 Risk of CLNM/LLNM

**Figure 2:**
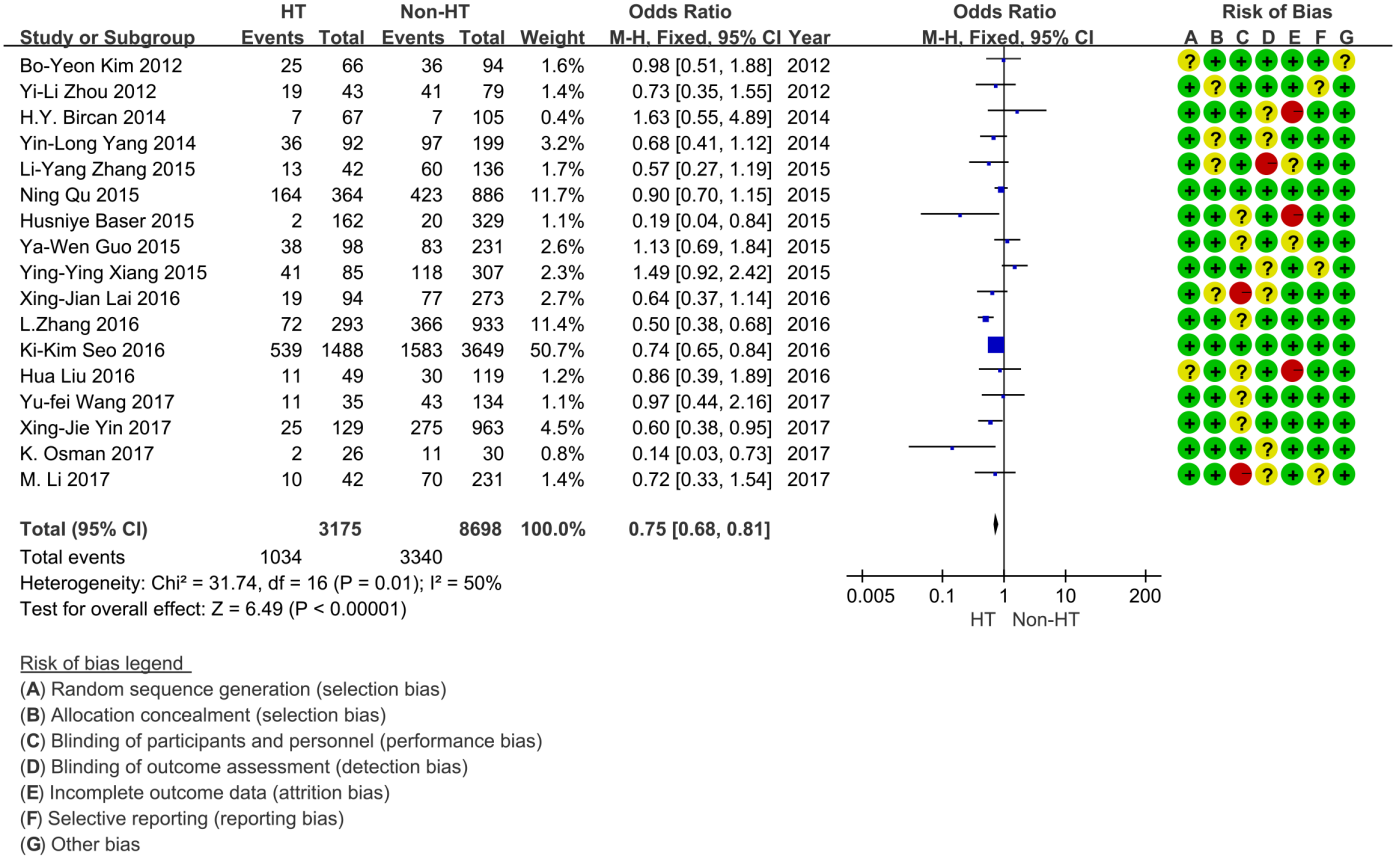
Forest plot for the risk of CLNM between HT and non-HT.

**Figure 3:**
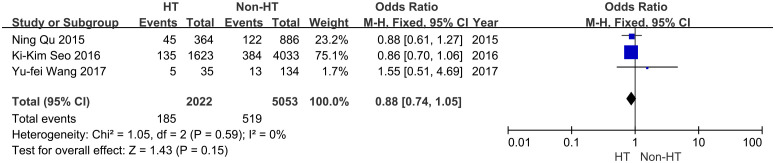
Forest plot for the risk of LLNM between HT and non-HT.

As a result, 32.6% (1,034/3,175) of PTMC patients with HT were diagnosed as CLNM. In contrast, 38.4% (3,340/8,698) of PTMC patients without HT developed CLNM. The risk of CLNM was lower in PTMC patients with coexisting HT (OR = 0.75, 95% CI: 0.68–0.81, *P* < 0.001) (**Figure 2**). Moderate heterogeneity was found in these studies (*I*^2^*=* 50%, *P* = 0.01). No publication bias was detected. Also, three studies evaluated LLNM status (29, 33, 35). There were 9.1% (185/2,022) of PTMC patients with HT who were diagnosed with LLNM, and 10.3% (519/5,053) of PTMC patients without HT developed LLNM. The risk of LLNM decreased significantly in PTMC patients with coexisting HT (OR = 0.88, 95% CI: 0.74–1.05, *P* = 0.15) (**Figure 3**). Low heterogeneity was found in these studies (*I*^2^ = 0%, *P* = 0.59).

### 3.3 Subgroup analysis between cN0 and uncertain cN0

**Figure 4:**
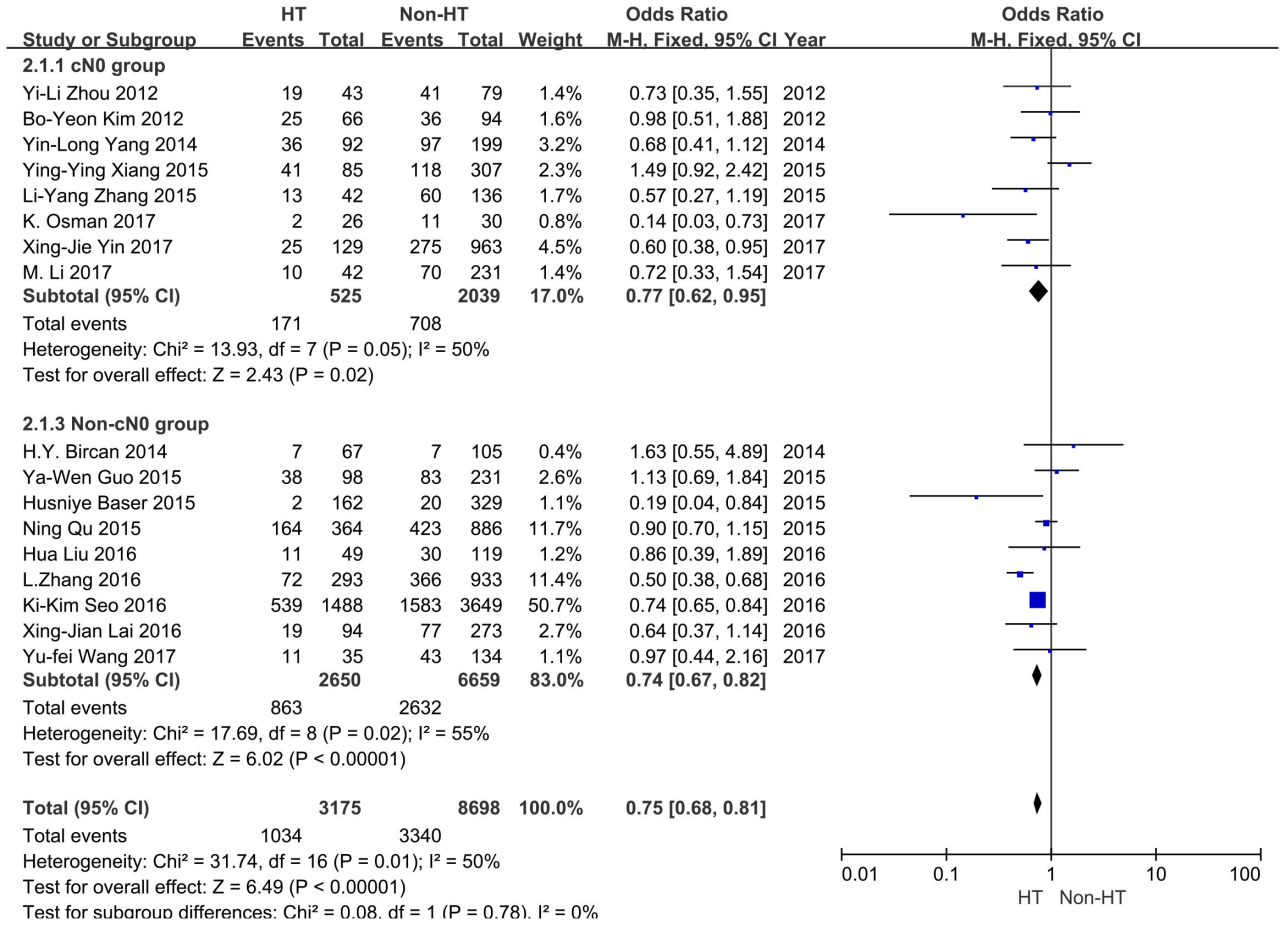
Forest plot for the cN0 and uncertain cN0 subgroups.

The meta-analyses included 2,564 PTMC patients with cN0 (525 patients with concurrent HT, 2039 patients without HT), involving six case–control and two cohort studies (21–23, 26, 31, 32, 34, 36). The included studies showed that 32.6% (171/525) of PTMC patients with HT had CLNM. IN contrast, 34.7% (708/2039) of PTMC patients with concurrent non-HT suffered CLNM. HT can decrease the risk of CLNM (OR 0.77, 95% CI: 0.62–0.95, *P* = 0.02) (**Figure 4**). Moderate heterogeneity was found between studies (*I*^2^ = 50%, *P* < 0.05). No publication bias was detected. Nine studies analyzed the correlation between HT and CLNM in the non-cN0 subgroup (20, 24, 25, 27–30, 33, 35).

### 3.4 Subgroup analysis in evaluation methods of CLN status

**Figure 5:**
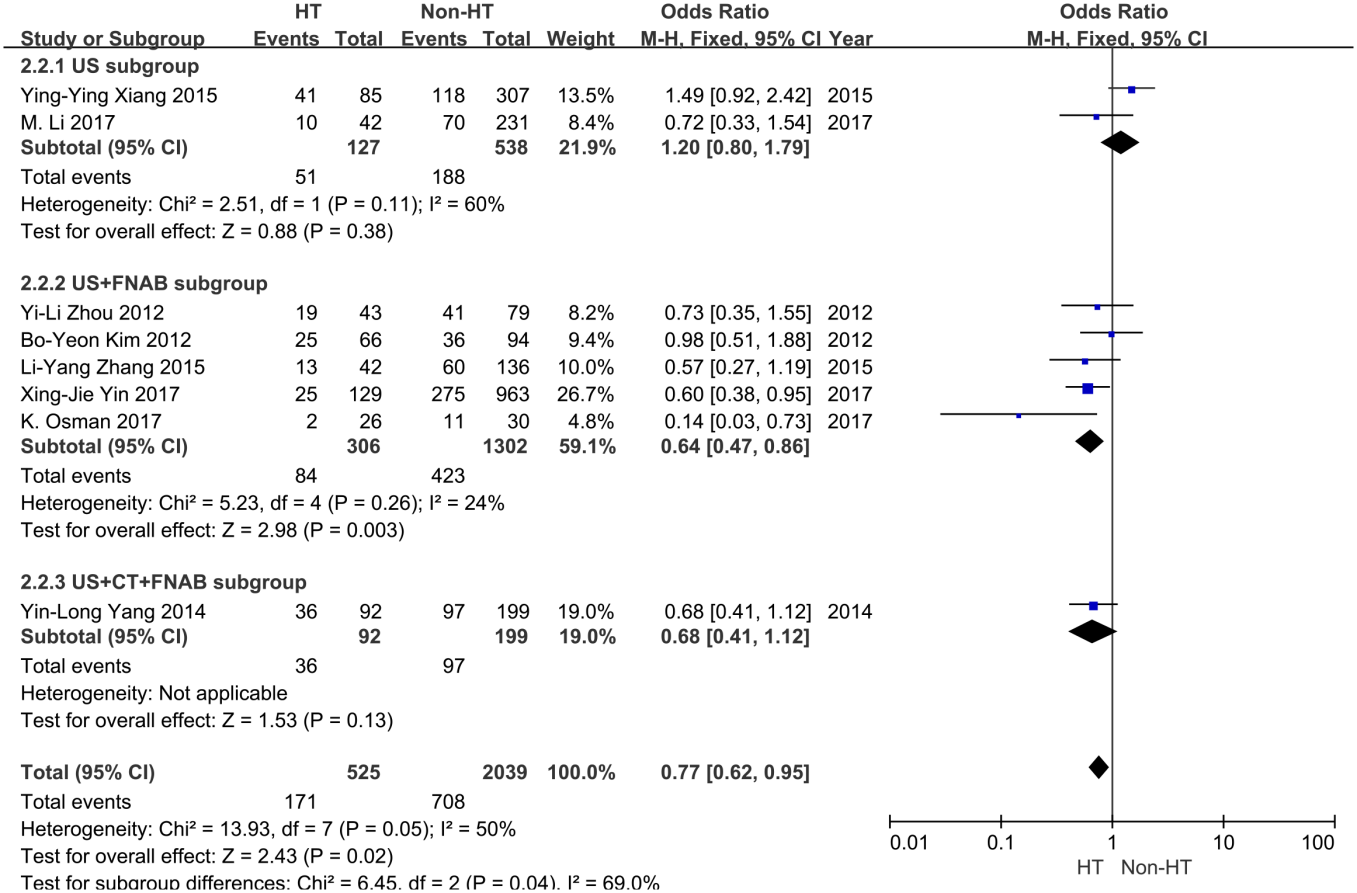
Forest plot to evaluate lymph node status using different methods.

Evaluation methods of LN status include palpation, preoperative ultrasonography (US), neck computed tomography (CT), and FNAB. Among the eight studies involving cN0 patients, two studies evaluated LN status using US (26, 36), five studies using US and FNAB (20, 21, 31, 32, 34), and one study using US, CT, and FNAB (23). In the US subgroup, 35.9% (239/665) of PTMC patients were diagnosed with CLNM. The outcome showed an association between HT and CLNM in this group (OR = 1.2, 95% CI: 0.8–1.79, *P* = 0.38). Moderate heterogeneity was found in between studies (*I*^2^ = 60%, *P* = 0.11) (**Figure 5**).

In the US and FNAB subgroup, 31.5% (507/1,608) of HT patients had CLNM. The result showed an association between HT and CLNM in this group (OR = 0.64, 95% CI: 0.47–0.86, *P* = 0.003) (**Figure 5**). No significant heterogeneity was found in between studies (*I*^2^ = 24% *P* = 0.26). In the CT and FNAB subgroup, 45.7% (133/291) of HT patients had CLNM. The result showed an association between HT and CLNM in this group (OR = 0.68, 95% CI: 0.41–1.12, *P* = 0.13) (**Figure 5**). Subgroup analysis was performed between the US subgroup, the US and FNAB subgroup, and the US, CT, and FNAB subgroup to evaluate whether the risk of CLN metastasis was statistically different using different methods of evaluating LN status. This result showed a statistical difference (*I*^2^ = 69%, *P* = 0.04).

### 3.5 Association between HT and clinicopathological parameters in the total and cN0 groups

Of the 303 patients diagnosed with PTMC, consisting of 227 women (74.9%) and 76 men (25.1%), there were 41 cases of PTMC patients with concurrent HT. The mean age at diagnosis was 41.5 ± 12 years in the HT group and 43.8 ± 12 years in the non-HT group (range 15 to 79 years). Univariate analyses showed a significant association between HT and gender, the total group (*P =* 0.002), and the cN0 group (*P* = 0.008). In contrast, there was no significant association between HT with age, tumor foci, ETE, tumor size, lymphadenopathy, TNM stage, and AJCC stage (**Table 1**).

### 3.6 Subanalysis examining CLN ≥5, between HT and PTMC clinicopathological parameters in the total and cN0 groups

In total, 178 patients with PTMC were identified as having undergone examination of the central lymph nodes, with at least five nodes assessed among the 154 cases classified as cN0. The subanalysis revealed a significant association between HT and positive CLNM in patients with five or more lymph nodes examined. Notably, PTMC patients with HT exhibited a relatively lower risk of CLNM compared to those without HT in the cN0 subgroup, with an odds ratio of 0.143, as shown in **Table 3**.

**Table 3:** Subanalysis of examined central lymph node ≥5 between HT and PTMC clinicopathological parameters in the total and cN0 groups.

Patients’ parameters	Total (178)	HT	Odds ratio	*P*-value	cN0 group (154)	HT	Odds ratio	*P*-value
	Non-HT				Non-HT			
Lymphadenopathy								
No	116	14	1	0.081	110	20	1	0.246
Yes	38	10	2.180 (0.895–5.312)		18	6	1.833 (0.648–5.185)	
CLNM								
No	70	15	1	0.120	60	17	1	0.085
Yes	84	9	0.500 (0.206–1.212)		68	9	0.467 (0.194–1.126)	
Positive lymph node (≥5)								
No	123	23	1	0.083	100	25	1	0.030*
Yes	31	1	0.173 (0.022–1.327)		28	1	0.143 (0.019–1.101)	

HT, Hashimoto’s thyroiditis; PTMC, papillary thyroid microcarcinoma; N0, clinically node-negative; CLNM, central lymph node metastasis.

*P < 0.05.

### 3.7 Neck lymph node and laboratory indications in PTMC patients with and without HT

In 270 PTMC patients, those with HT had a higher number of examined CLN than those without HT (*P* = 0.007). Among 108 cases of PTMC, HT patients had a lower number of CLNM and a lower CLNM rate than those without HT (*P* = 0.015 and *P* < 0.001, respectively). Of 257 PTMC patients, HT patients had elevated TPOAb and TgAb levels than those without HT (*P* < 0.0001 and *P =* 0.012, respectively). The number of LLN examined, the number of LLNM, the LLNM rate, and TSH were not significantly different between groups with and without HT (**Table 4**).

**Table 4:** Central/lateral neck lymph node examined/metastasis and laboratory indications in PTMC patients with and without HT.

Patients’ parameters	Total (PTMC)	Non-HT	HT	*P*-value
Number of examined CLN	270	5.9 ± 4.5	8.3 ± 6.0	0.0074*
Number of CLNM	108	3.7 ± 3.5	2.1 ± 1.8	0.0150*
Rate of CLNM (%)	108	56.2 ± 30.3	28.0 ± 13.8	<0.0001*
Number of examined LLN	27	20.8 ± 11.5	27.5 ± 6.4	0.4330
Number of LLNM	22	7.3 ± 9.0	3.0 ± 2.8	0.5230
Rate of LLNM (%)	22	35.0 ± 27.3	10.0 ± 8.5	0.2220
TPOAb	257	47.5 ± 116.3	200.2 ± 198.6	<0.0001*
TgAb	257	63.5 ± 290.0	432.6 ± 790.7	0.0120*
TSH	257	1.7 ± 2.2	5.6 ± 21.0	0.2850

PTMC, papillary thyroid microcarcinoma; HT, Hashimoto’s thyroiditis; CLN, central neck lymph node; CLNM, central neck lymph node metastasis; LLN, lateral neck lymph node; LLNM, lateral neck lymph node metastasis.

*P < 0.05.

### 3.8 TPOAb and TgAb for CLNM in 257 patients

**Figure 6:**
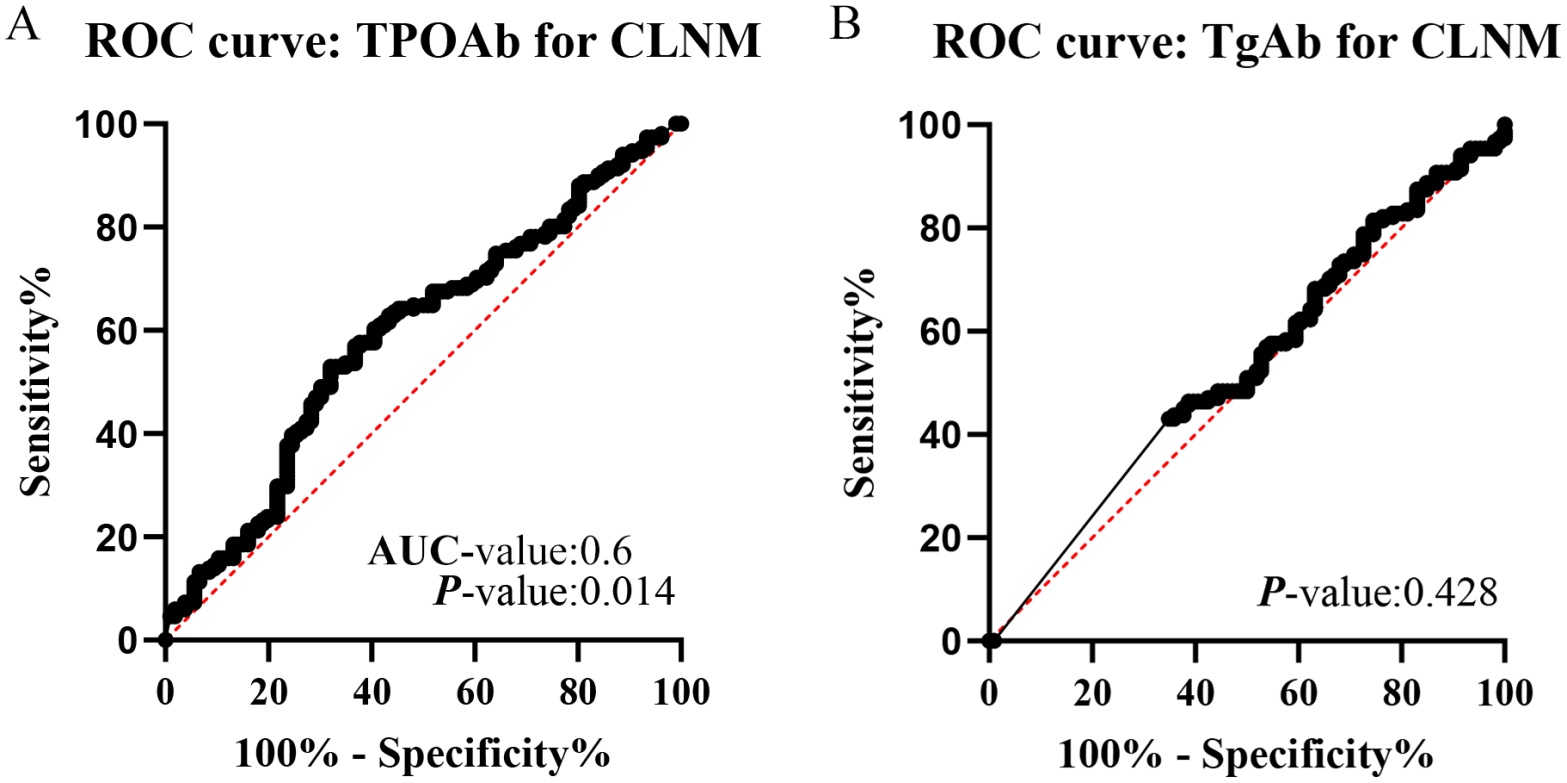
ROC curve of TPOAb for CLNM **(A)** and TgAb for CLNM **(B)**.

The predictive ability of TPOAb for CLNM was assessed using ROC curves. The fitted ROC area under the curve (AUC) was 0.6, indicating a modest discriminatory power. The sensitivity and specificity at the optimal cutoff value (17.09 IU/mL, determined by the Youden index) were 52.98% and 67.92%, respectively (**Figure 6**). Given that an AUC of 0.5 represents a chance-level prediction, these results suggest that TPOAb alone has limited efficacy as a standalone predictor for CLNM. Conversely, the predictive ability of TgAb for CLNM was poor. These PTMC cases were separated into the TPOAb ≤17.09 group and the TPOAb >17.09 group based on this cutoff value. Consistent with the ROC findings, the TPOAb >17.09 group had a significantly lower number of CLNM and rate of CLNM than the TPOAb ≤17.09 group (**Supplementary Figure S2**), supporting its role as a correlative rather than a strongly predictive biomarker.

### 3.9 Publication bias

**Figure 7:**
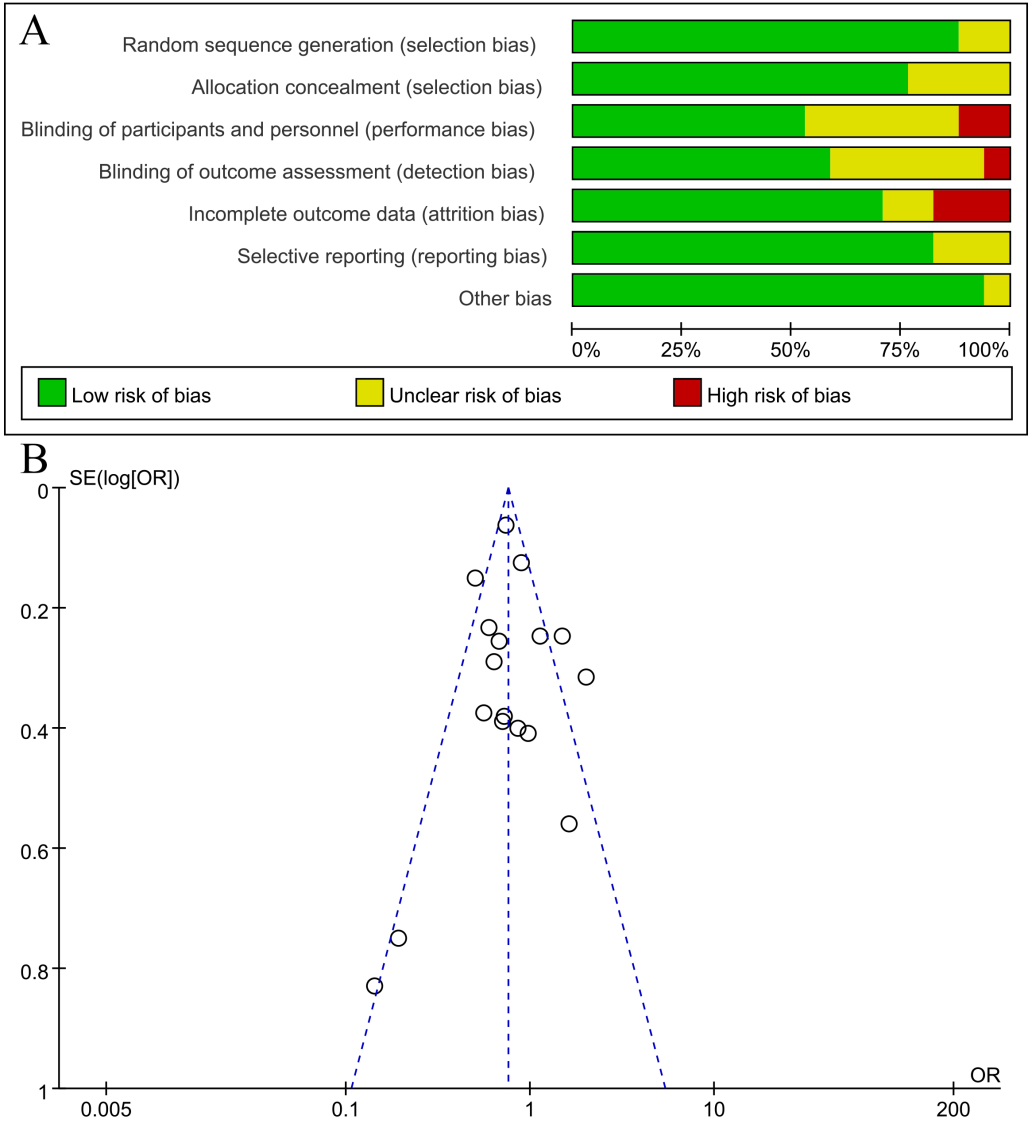
Funnel plot for publication bias **(A)** and risk bias **(B)**.

Begg’s funnel plot was used to evaluate publication bias. As shown in **Figures 7**, S1, no publication bias was found.

### 3.10 Sensitivity analysis

To assess the robustness of our primary findings, sensitivity analysis was performed by excluding the three studies with low-quality scores (NOS = 5) (**Table 2**). The results remained consistent with the original meta-analysis.

After excluding these studies, the pooled data from the remaining 14 studies (involving 10,222 PTMC patients) continued to show a significantly lower risk of CLNM in the HT group compared to the non-HT group. The recalculated OR was 0.76 (95% CI: 0.69–0.84, *P* < 0.001), which is nearly identical to the original OR of 0.75. Heterogeneity decreased slightly from *I*^2^ = 50% to *I*^2^ = 45%.

This sensitivity analysis confirms that the protective association between Hashimoto’s thyroiditis and central lymph node metastasis in PTMC is robust and not unduly influenced by lower-quality studies.

## 4 Discussion

Recently, the incidence of PTMC concurrent with HT has significantly increased. Whether prophylactic CLND needs to be performed in cN0 PTMC patients has been controversial for a long time. Prophylactic CLND has the potential to reduce recurrence and mortality rates in PTMC patients. International guidelines do not routinely recommend this procedure for cN0 PTMC patients due to the associated risks and complications, particularly in those with HT, who often experience higher rates of postoperative complications (37, 38). According to international guidelines, prophylactic CLND is not routinely recommended for cN0 PTMC (39). HT patients commonly have a higher prevalence rate of postoperative complications (40). Simultaneously, preoperative evaluation of CLN status in PTMC patients by imaging examination is limited (41). This study evaluates the risk of neck LNM in cN0 PTMC patients with coexisting HT to guide optimal surgical decision-making.

Initially, in the meta-analysis part, the study demonstrated that PTMC patients with coexisting HT had a meaningfully lower risk of CLN metastasis than patients without HT. Although the underlying mechanism by which HT affects PTMC remains unclear, some hypotheses have been proposed. Chronic inflammation-induced carcinoma has been regarded as one of the possible mechanisms (42). Moreover, the present study proposes that cancer cells may be destroyed by autoimmunity, specifically through thyroid-specific antigens targeting the cancer cells (43). Simultaneously, some studies found that HT appeared to act as a protective factor in univariate analyses (44–46). HT is characterized by chronic inflammation, which causes dense fibrosis in the thyroid gland, and fibrosis may inhibit PTC development and progression (47–49). Available results remain limited and somewhat discordant (22, 25, 26). The probable reason for this difference may be due to a higher proportion of male patients and multifocal tumors in these studies, which are associated with a higher LNM risk (50).

HT is characterized by chronic inflammation, often resulting in dense fibrosis within the thyroid gland. This fibrotic process may inhibit the development and progression of PTC, potentially leading to a more favorable prognosis in some patients. However, existing results are limited and show discordance, possibly due to the higher representation of male patients and multifocal disease in certain studies, both of which are associated with an increased risk of LNM (51, 52).

Furthermore, a subgroup analysis was conducted comparing the cN0 group to those with uncertain cN0 status to evaluate differences in the risk of CLN metastasis. This analysis revealed that combining ultrasound with FNAB provided a more reliable assessment of lymph node status than ultrasound alone (53, 54). Focal lymphocytic thyroiditis in HT may be misinterpreted as suspicious nodules on ultrasound, underscoring the necessity for precise diagnostic techniques (55). Consequently, the integration of ultrasound and ultrasound-guided FNAB is valuable for improving diagnostic accuracy in this patient population (56, 57). To further support these findings, additional research is needed to explore the molecular mechanisms underlying the interaction between HT and PTC progression, particularly in the context of inflammation and fibrosis. Understanding these pathways could lead to the identification of novel biomarkers that enhance patient stratification and guide therapeutic interventions.

Concomitant regional lymphadenopathy is a common comorbidity in the thyroid gland surrounding HT (40). Further retrospective analysis reveals that the examined rate of LN was higher in PTMC patients with HT compared to patients with solitary PTMC, consistent with previous findings. However, the former has a lower positive risk of LNM than the latter. Meanwhile, HT has a lower risk of positive CLNM (≥5). We believe that TPOAb was likely a protective prognostic factor. PTMC patients with concurrent HT and TPOAb >17.09 IU/mL had a lower risk of CLNM compared to those with TPOAb ≤17.09 IU/mL.

In contrast, previous studies have reported that PTC tumors with HT are more likely to be bilateral and multifocal and have a higher stage disease and a greater frequency of LN metastasis (11, 58, 59). Interestingly, our data demonstrated that the frequency of LN metastasis was significantly greater in PTMC patients with low TPOAb (≤17.09) than those with high TPOAb (>17.09). These data indicate that thyroiditis immunity may play an essential role in reducing LNM in PTMC patients. However, it is found that the effect of HT does not significantly lower the risk of LLNM. The results are consistent with previous findings (60). Our current research cannot explain why HT disease has decreased the risk of CLNM, but does not affect LLNM.

Although it was hard to identify cN0 patients with coexisting HT using US and FNAB, our results demonstrated a lower risk of CLNM in these patients. Moreover, some studies held that prophylactic CLND does not improve long-term regional disease control or survival, regardless of the pathological status of LN retrieved (61). Also, prophylactic CLND prolongs the operation time and increases postoperative complications. Therefore, when PTMC coexists with HT and preoperative certain cN0, the extent of surgery can be performed relatively conservatively, and patients may get more benefit than prophylactic CLND.

This study has several limitations that should be acknowledged. First, the retrospective design of both the meta-analysis and the case–control component introduces inherent biases, such as selection and information bias, which may affect the generalizability of our findings. Second, the included studies exhibited significant heterogeneity in terms of patient demographics, surgical procedures, and diagnostic criteria for HT and CLNM, which could influence the pooled estimates. Third, the methods for detecting TPOAb levels varied across studies, potentially leading to inconsistencies in the cutoff values and their clinical interpretation. Most importantly, as indicated by our ROC analysis (AUC = 0.6), the predictive power of TPOAb alone for CLNM is limited, approaching chance level. Therefore, TPOAb should not be used in isolation for clinical decision-making but rather interpreted as a supportive biomarker within the broader clinical context.

Despite these limitations, our findings have important implications for future clinical practice. The identification of HT as a protective factor against CLNM in PTMC provides a valuable element for risk stratification. While TPOAb alone is a suboptimal predictor, its significant association with reduced CLNM risk suggests its potential utility as part of a multiparameter assessment. To build upon our findings and directly address the current limitation of standalone biomarker performance, future research should prioritize the development of integrated predictive models. As suggested by the reviewer, constructing a model that incorporates TPOAb levels alongside other crucial variables—such as ultrasonographic features, tumor size, multifocality, and patient demographics—could significantly enhance preoperative risk assessment. Advanced statistical methods, including machine learning algorithms applied to large, multi-institutional cohorts, would be ideal for this purpose. Such a model would move beyond singular biomarkers and offer a clinically robust tool for personalizing the management of cN0 PTMC patients, particularly in deciding the necessity of prophylactic CLND. Future prospective studies with standardized protocols are needed to validate these results and establish such comprehensive predictive tools.

## 5 Limitation

This study has several limitations. First, the retrospective nature of both the meta-analysis and the case–control study introduces potential biases, including selection bias and confounding factors, which may affect the validity of the results. Second, there was considerable heterogeneity among the included studies in terms of study design, patient characteristics, surgical techniques, and diagnostic criteria for HT and lymph node metastasis. Although we used random-effects models to address this, the variability may still impact the generalizability of our findings. Third, the methods for measuring TPOAb levels were not standardized across studies, leading to potential inconsistencies in the cutoff values and their clinical applicability. Future prospective studies with uniform protocols are necessary to confirm our conclusions and establish reliable biomarkers for clinical use.

## 6 Conclusion

The treatment of PTMC with coexisting HT presents unique challenges and opportunities for personalized medicine. Our study highlights the complex interplay between HT and LNM in PTMC patients, emphasizing the need for a tailored approach in clinical decision-making.

Our findings indicate that patients with PTMC and elevated TPOAb levels (>17.09 IU/mL) exhibit a significantly lower risk of CLNM compared to those with lower TPOAb levels (62, 63). This suggests that TPOAb may serve as a protective prognostic factor (64). The integration of predictive models, such as those developed using advanced machine learning techniques, can enhance the accuracy of prognostic assessments, allowing for early intervention strategies tailored to individual patient profiles (65, 66).

The study identified specific molecular characteristics of endothelial cells associated with drug resistance at the single-cell level. These findings suggest that targeting these molecular pathways could provide new avenues for preventing drug resistance in PTMC patients. By employing multi-omics clustering algorithms, clinicians can categorize patients based on their unique biological features, enabling the development of preventive strategies that mitigate adverse reactions during treatment (67–69).

Our research underscores the importance of personalizing treatment plans based on the distinct characteristics of different PTMC subtypes. The identification of four subtypes, each with varying responses to targeted therapies, highlights the potential for tailored treatment approaches. For instance, patients classified under the CS2 subtype, which shows a greater sensitivity to anti-angiogenic agents, can be prioritized for such therapies, while those in the CS3 subtype may benefit from treatments targeting phosphorylation pathways (70–72).

In summary, this study provides evidence that HT reduces the risk of CLNM in PTMC, with TPOAb serving as a potential biomarker for risk stratification. Insights support the development of more conservative surgical strategies in selected patients, reducing unnecessary interventions and improving quality of life. Future work should focus on validating these findings in prospective settings and integrating multi-omics data to further refine personalized management strategies for PTMC.

## Data Availability

The original contributions presented in the study are included in the article/**Supplementary Material**. Further inquiries can be directed to the corresponding authors.

## Supplementary material

Supplementary Figure 1 *Begg’s* funnel plot was used to evaluate publication bias.

Supplementary Figure 2 The different analyses of between in TPOAb ≤ 17.09 group and TPOAb>17.09 group. **(A)** Number of examined CLN; **(B)** Number of CLNM; **(C)** Rate of CLNM (%).

Supplementary Table 1 The subanalysis of examined CLN ≥ 5, between HT and PTMC clinicopathological parameters in total group and cN0 group. ^*^*P*<0.05.

Supplementary Table 2 Some central/lateral neck lymph node examined/metastasis, and laboratory indications in PTMC patients with and without HT. ^*^*P*<0.05.
