# Supplementary figures and images for: Hashimoto’s thyroiditis reduces central lymph node metastasis risk in papillary thyroid microcarcinoma: an integrated meta-analysis
Source: Front Endocrinol (Lausanne). 2025 Nov 24;16:1695508. doi: 10.3389/fendo.2025.1695508 (PMC12682656; doi:10.3389/fendo.2025.1695508)

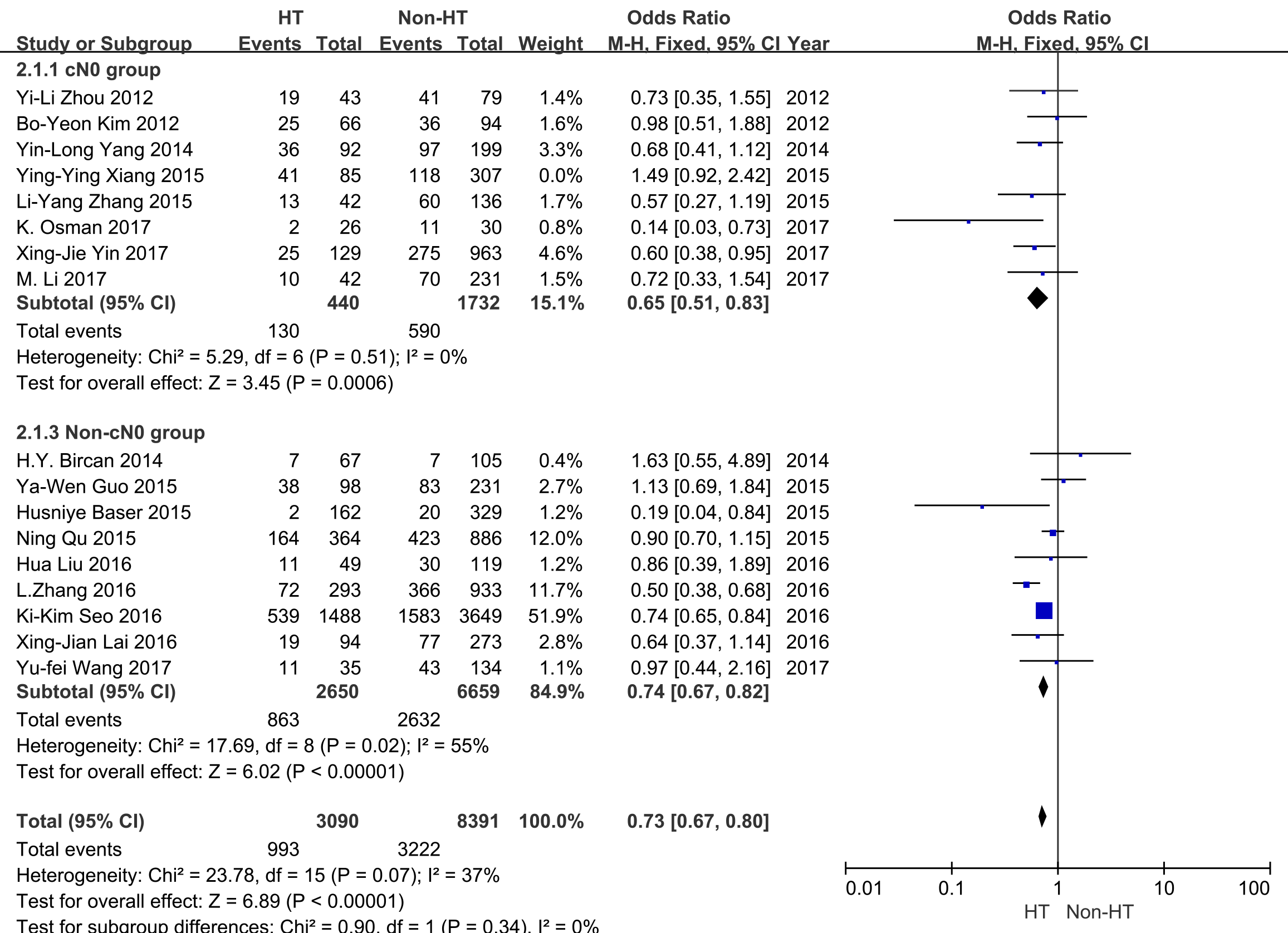

Supplement: Supplementary Figure 1 — Begg’s funnel plot was used to evaluate publication bias. [file Image1.tif]
