# Supplementary material for: Hashimoto’s thyroiditis reduces central lymph node metastasis risk in papillary thyroid microcarcinoma: an integrated meta-analysis
Source: Front Endocrinol (Lausanne). 2025 Nov 24;16:1695508. doi: 10.3389/fendo.2025.1695508 (PMC12682656; doi:10.3389/fendo.2025.1695508)
Supplement: Supplementary Table 1 — The subanalysis of examined CLN ≥ 5, between HT and PTMC clinicopathological parameters in total group and cN0 group. *P<0.05. [file Table1.docx]

**Table S1** The subanalysis of examined central lymph-node ≥5, between HT and PTMC clinicopathological parameters in total group and cN0 group.

| **Patients’ parameters** | **Total (178)** |  |  |  |  | **cN0 group**  **（154）** |  |  |  |
| --- | --- | --- | --- | --- | --- | --- | --- | --- | --- |
|  | **Non-HT** | **HT** | **Odds Ratio** | ***P* value** |  | **Non-HT** | **HT** | **Odds Ratio** | ***P* value** |
| **Lymph-adenopathy** |  |  |  |  |  |  |  |  |  |
| No | 116 | 14 | 1 | 0.081 |  | 110 | 20 | 1 | 0.246 |
| Yes | 38 | 10 | 2.180(0.895-5.312) |  |  | 18 | 6 | 1.833(0.648-5.185) |  |
| **CLNM** |  |  |  |  |  |  |  |  |  |
| No | 70 | 15 | 1 | 0.120 |  | 60 | 17 | 1 | 0.085 |
| Yes | 84 | 9 | 0.500(0.206-1.212) |  |  | 68 | 9 | 0.467(0.194-1.126) |  |
| **Positive of**  **Lymph-node (**≥5**)** |  |  |  |  |  |  |  |  |  |
| No | 123 | 23 | 1 | 0.083 |  | 100 | 25 | 1 | 0.030* |
| Yes | 31 | 1 | 0.173(0.022-1.327) |  |  | 28 | 1 | 0.143(0.019-1.101) |  |

**Abbreviations:** HT: hashimoto’s thyroiditis; PTMC: papillary thyroid microcarcinoma; N0: clinically node-negative；CLNM: central lymph node metastasis; *: P＜0.05.
