# Supplementary material for: Hashimoto’s thyroiditis reduces central lymph node metastasis risk in papillary thyroid microcarcinoma: an integrated meta-analysis
Source: Front Endocrinol (Lausanne). 2025 Nov 24;16:1695508. doi: 10.3389/fendo.2025.1695508 (PMC12682656; doi:10.3389/fendo.2025.1695508)
Supplement: Supplementary Table 2 — Some central/lateral neck lymph node examined/metastasis, and laboratory indications in PTMC patients with and without HT. *P<0.05. [file Table2.docx]

**Table S2** Some central/lateral neck lymph node examined/metastasis, and laboratory indications in PTMC patients with and without HT.

| **Patients’ parameters** | **Total (PTMC)** | **Non-HT** | **HT** | ***P* value** |
| --- | --- | --- | --- | --- |
| Number of examined CLN | 270 | 5.9±4.5 | 8.3±6.0 | 0.0074* |
| Number of CLNM | 108 | 3.7±3.5 | 2.1±1.8 | 0.0150* |
| Rate of CLNM (%) | 108 | 56.2±30.3 | 28.0±13.8 | ＜0.0001* |
| Number of examined LLN | 27 | 20.8±11.5 | 27.5±6.4 | 0.4330 |
| Number of LLNM | 22 | 7.3±9.0 | 3.0+2.8 | 0.5230 |
| Rate of LLNM (%) | 22 | 35.0±27.3 | 10.0±8.5 | 0.2220 |
| TPOAb | 257 | 47.5±116.3 | 200.2±198.6 | ＜0.0001* |
| TgAb | 257 | 63.5±290.0 | 432.6±790.7 | 0.0120* |
| TSH | 257 | 1.7±2.2 | 5.6±21.0 | 0.2850 |

**Abbreviations:** PTMC: papillary thyroid microcarcinoma; HT: hashimoto’s thyroiditis; CLN: central neck lymph node; CLNM: central neck lymph node metastasis; LLN: lateral neck lymph node; LLNM: lateral neck lymph node metastasis; *: P＜0.05.
